# Prediction and analysis of Corona Virus Disease 2019

**DOI:** 10.1371/journal.pone.0239960

**Published:** 2020-10-05

**Authors:** Yan Hao, Ting Xu, Hongping Hu, Peng Wang, Yanping Bai

**Affiliations:** 1 School of Information and Communication Engineering, North University of China, Taiyuan, China; 2 Department of Mathematics, School of Science, North University of China, Taiyuan, China; Western Norway University of Applied Sciences, NORWAY

## Abstract

The outbreak of Corona Virus Disease 2019 (COVID-19) in Wuhan has significantly impacted the economy and society globally. Countries are in a strict state of prevention and control of this pandemic. In this study, the development trend analysis of the cumulative confirmed cases, cumulative deaths, and cumulative cured cases was conducted based on data from Wuhan, Hubei Province, China from January 23, 2020 to April 6, 2020 using an Elman neural network, long short-term memory (LSTM), and support vector machine (SVM). A SVM with fuzzy granulation was used to predict the growth range of confirmed new cases, new deaths, and new cured cases. The experimental results showed that the Elman neural network and SVM used in this study can predict the development trend of cumulative confirmed cases, deaths, and cured cases, whereas LSTM is more suitable for the prediction of the cumulative confirmed cases. The SVM with fuzzy granulation can successfully predict the growth range of confirmed new cases and new cured cases, although the average predicted values are slightly large. Currently, the United States is the epicenter of the COVID-19 pandemic. We also used data modeling from the United States to further verify the validity of the proposed models.

## Introduction

Infectious diseases are caused by various pathogens that can be transmitted from person to person, animal to animal, or person to animal. They can be transmitted in various ways, and the speed of transmission is fast. Early diagnosis of infectious diseases is crucial, and prevention and control are paramount.

In December 2019, an unexplained viral pneumonia was reported in Wuhan, China. The virus, named the 2019 Novel Coronavirus (2019-nCoV) by the World Health Organization (WHO) on January 12, 2020, causes Corona Virus Disease 2019 (COVID-19), and is currently the seventh known species of coronavirus that can infect human beings. The remaining six species are human coronavirus (HCoV)-229E, HCoV-OC43, HCoV-NL63, HCoV-HKU1, severe acute respiratory syndrome (SARS)-CoV, and Middle East respiratory syndrome (MERS)-CoV. COVID-19 and severe acute respiratory syndrome (SARS), which broke out in 2003, are both caused by coronaviruses. Their clinical symptoms are similar but not identical. People infected with the 2019-nCoV will experience varying symptoms, including fever, mild cough, or pneumonia, occasionally leading to death. The mortality rate of COVID-19 is approximately 2% to 4%, although this is an extremely early percentage and may change as more information becomes available. Meanwhile, this does not mean that the virus is not serious, it simply means that not everyone infected with it will face the worst outcome. We are currently in a tense state of prevention and control, and are concerned about a global pandemic.

2019-nCoV is an intractable and unexpected virus with the following characteristics. First, it has a strong ability to camouflage itself. The virus can be present asymptomatically, making an infected person appear healthy, or can manifest with various other symptoms in which an infected person seems to be suffering from a common respiratory disease. It also has a long latency period. According to a report from Lancet, the median latency time of the new coronavirus is approximately 20 days, reaching up to 37 days for some patients. In addition, it is transmitted in various ways. The transmission method of the 2019-nCoV is similar to that of various other infectious diseases. In addition to a traditional droplet transmission, it also includes contact, air, fecal–oral, and blood transmissions. A high relapse rate has also been seen. Since the initial outbreak of COVID-19, there have been many cases of recovered patients testing positive again after a reexamination, even leading to death after a relapse. The virus has also shown variability. As a foreboding aspect of the future development of novel coronaviruses, their rapid genetic recombination results in a mutation into new strains. Each recombination can increase the toxicity and infectivity, invalidating original treatment methods and drugs. Forty variants of novel coronaviruses have been discovered by scientists in Iceland. Finally, it is highly infectious. Scientific data have shown that the affinity between the S-protein of 2019-nCoV and angiotensin converting enzyme 2 (ACE2) is 10 to 20 times that of SARS, which means that the infectivity of COVID-19 is significantly higher than that of SARS.

There are currently no specific treatments for COVID-19. However, many symptoms can be treated, and treatment must be given according to the clinical status of the patient. In addition, supplementary care for those infected may be effective. Self-protection includes maintaining basic hand and respiratory hygiene, adhering to safe eating habits, and avoiding close contact with anyone who shows symptoms of a respiratory disease (such as coughing or sneezing). At present, owing to the widespread nature of COVID-19, factories are being shut down, schools are being suspended, and people are isolating in their own homes, significantly disrupting daily life. It is therefore extremely important to reasonably predict and analyze the development trend of this pandemic.

In previous studies, the prediction methods for the occurrence, spread, and change in infectious diseases mainly included regression prediction models [1, 2], Markov chain models [3], Bayesian networks [4–6], and other machine learning methods [7–9]. Most of the previous studies have been based on research related to influenza, with a few studies conducted on human immunodeficiency virus (HIV) infection [10, 11] and SARS [12, 13].

For example, Ray [14] used ensemble methods with three component models to predict the seasonal timing of influenza and severity in the United States. Xue et al. [15] established five models for predicting and assessing influenza outbreaks across 10 regions of the United States. Experiments on Google Flu Trends (GFT) and Centers for Disease Control (CDC) data have shown that GFT and historical influenza data are partially complementary and that GFT+CDC regression is a preferable model. In addition, Alkouz et al. [16] proposed a system called Tweetluenza, which can predict the spread of influenza in real time.

Many scholars have published reports on COVID-19. For example, in [17–20], its clinical features are described, and in [19] and [20], the authors demonstrated that COVID-19 has a high comorbidity with hypertension, diabetes, and cardiovascular diseases. Anastassopoulou et al. [21] estimated the case fatality and case recovery ratios based on a susceptible infectious-recovered-dead (SIDR) model, along with their 90% confidence intervals as the outbreak evolves. In [22–27], the authors use machine learning methods to diagnose COVID-19 based on X-ray and CT images. Randhawa et al. [28] also used machine learning-based methods to achieve ultra-fast, scalable, and highly accurate classification of whole COVID-19 virus genomes based on the COVID-19 intrinsic virus genomic signature. In [29–31], other characteristics related to COVID-19 were studied.

To date, few studies related to the use of quantitative prediction methods to predict the development trend and growth range of COVID-19 have been conducted. In the present study, three methods, namely, an Elman neural network, LSTM, and SVM are applied to predict and analyze COVID-19 data from January 23, 2020 to April 6, 2020 in Wuhan, Hubei Province, China, including cumulative confirmed cases, confirmed new cases, cumulative deaths, new deaths, and cumulative cured cases and new cured cases. First, the Elman neural network, LSTM, and SVM were used to predict the development trend of cumulative confirmed cases, cumulative deaths, and cumulative cured cases. Next, the SVM with fuzzy granulation was used to predict the growth range of confirmed new cases, new deaths, and new cured cases. Experimental results showed that the Elman neural network and SVM adopted in this study can accurately predict the development trend of COVID-19, whereas LSTM is more suitable for the prediction of cumulative confirmed cases. The SVM with fuzzy granulation is effective for the growth range prediction of confirmed new cases, new deaths, and new cured cases, despite the larger averages. The same models were also used on data in the United States to verify the robustness of the models.

The rest of this paper is organized as follows. Section 2 introduces the methods applied. Section 3 describes the experiments and analysis results. Finally, some concluding remarks and areas of future research are provided in Section 4.

## Methods

There are two main research topics in this paper. The first is the development trend prediction and analysis of the cumulative confirmed cases, deaths, and cured cases. The second is the growth range prediction of the confirmed new cases, new deaths, and new cured cases. Because the present state of cumulative confirmed cases, deaths, and cured cases is related to the state of the previous day, and shows a non-linear growth, recurrent neural networks (RNNs) are applicable. In this study, we mainly apply the Elman neural network and LSTM. In addition, the SVM has a better prediction effect on non-linear data, therefore, the SVM model is also introduced for development trend prediction. Because there are many factors affecting the growth of new cases, the growth trend fluctuates significantly. Here, only the growth range of the new cases is predicted. To predict the change space of the three datasets more accurately, this paper introduces a SVM model with fuzzy granulation.

### A. Elman neural network

**Fig 1:**
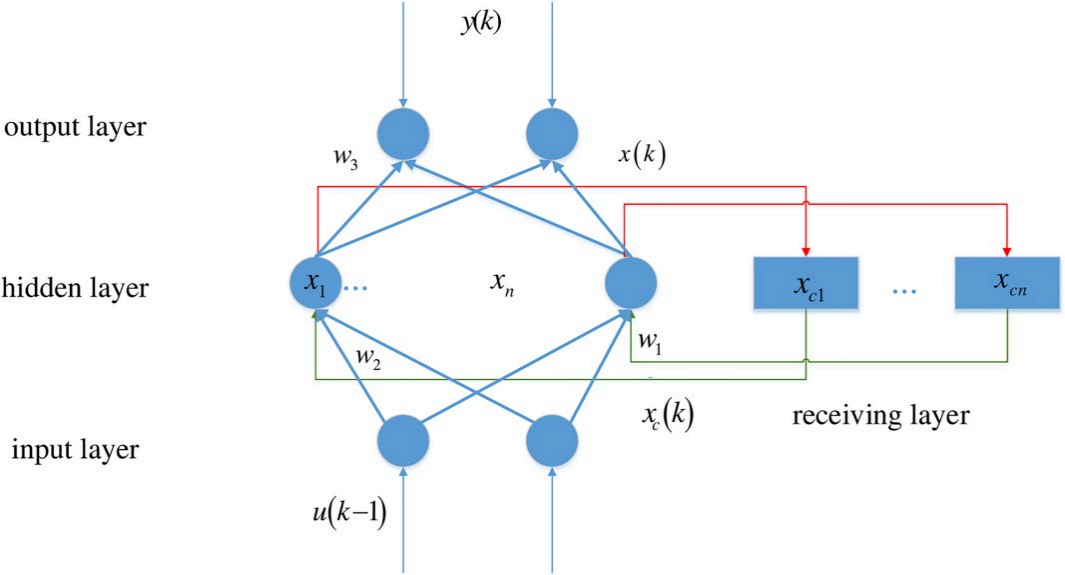
Network structure of the Elman neural network.

The Elman neural network is generally divided into four layers: input, hidden, receiving, and output layers. The receiving layer is also called the context layer or state layer, and is used to memorize the output value of the hidden layer unit at the previous moment and return it to the network input. It can be considered a one-step delay operator. As the characteristic of the Elman neural network, the output of the hidden layer is self-connected to the input of the hidden layer through the delay and storage of the receiving layer. This self-connected method makes it sensitive to historical data. The addition of an internal feedback network enhances the ability of the network to process dynamic information, thereby achieving the purpose of dynamic modeling. The network structure of the Elman neural network is shown in Fig 1.

The connections of the input layer, hidden layer, and output layer are similar to those of a feedforward network. Unlike a feedforward network, the Elman neural network contains a receiving layer, which takes the hidden layer state of the previous moment together with the network input of the current moment as the input of the hidden layer.

Here, *x*_*c*_(*k*) = *x*(*k* − 1) indicates that the feedback state vector is equal to the hidden layer state at the previous moment. The hidden layer state is expressed as follows:

$$x(k)=f(w_{1}x_{c}(k)+w_{2}(u(k-1))).$$

The output layer state is expressed as

$$y(k)=g(w_{3}x(k)),$$

where *u* is the input vector, *x*_*c*_ is the feedback state vector, *w*_1_ is the weight of the receiving layer to the hidden layer, *w*_2_ is the weight of the input layer to the hidden layer, *w*_3_ is the weight of the hidden layer to the output layer, *f*(*) is the activation function of the hidden layer, and *g*(*) is the activation function of the output layer.

### B. Long short-term memory

**Fig 2:**
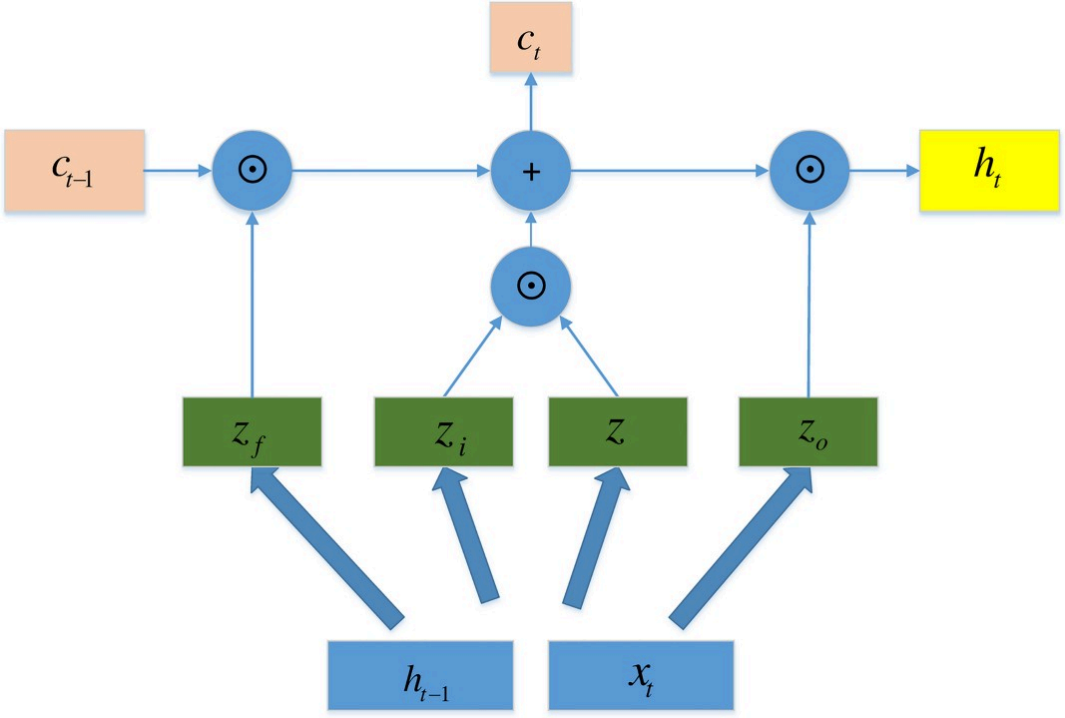
Diagram of the LSTM.

LSTM networks are variants of RNNs. General RNNs only have short-term memory owing to a disappearance of the gradients. LSTM networks combine short-term and long-term memory through a subtle gate control and solve the problem of disappearing gradients to a certain extent. LSTM was proposed by Hochreiter and Schmidhuber [32] in 1997. It has three special structures: a forget gate, an input gate, and an output gate. LSTM networks can delete or add information to a cell state through a structure called a gate. Fig 2 shows a diagram of the LSTM network.

The diagram shows the details of the forget gate, input gate, and output gate. The first step is to decide what information to discard from the cell state, which is done through a layer called the forget gate. The forget gate reads the hidden state of the previous moment *h*_*t*−1_ and the current input data *x*_*t*_, and then outputs a vector between zero and 1. The value between zero and 1 in this vector indicates how much information is retained or discarded in the cell state *c*_*t*_. A value of zero means all information is discarded, and 1 means all information is retained.

$$z_{f}=\sigma (W_{f}\cdot [h_{t-1},x_{t}]+b_{f}).$$

The next step is to decide how much new information is added to the cell state. Two steps are needed to achieve this. First, we use *h*_*t*−1_ and *x*_*t*_ to decide which information to update through an input gate operation. We then use *h*_*t*−1_ and *x*_*t*_ to obtain a new candidate cell state *z* through a tanh layer.

$$z_{i}=\sigma (W_{i}\cdot [h_{t-1},x_{t}]+b_{i}),$$

$$z=\text{tanh}(W\cdot [h_{t-1},x_{t}]+b).$$

Next, the cell state is updated as follows:

$$c_{t}=z_{f}⊙c_{t-1}+z_{i}⊙z.$$

Finally, we need to determine the output value. After updating the cell state, we need to determine which part of the cell state will be output according to the input *h*_*t*−1_ and *x*_*t*_. Here, we need to pass the input through a sigmoid layer called the output gate to obtain the judgment conditions. We then need to pass the cell state through the tanh layer to obtain a vector between -1 and 1, which is multiplied by the judgment conditions obtained by the output gate to obtain the output.

$$z_{o}=\sigma (W_{o}\cdot [h_{t-1},x_{t}]+b_{o}),$$

$$h_{t}=z_{o}⊙\text{tanh}(c_{t}),$$

where *z*_*i*_, *z*_*f*_, and *z*_*o*_ are the gate control state of the forget gate, input gate, and output gate, respectively. In addition, *z* is the input through a tanh layer, which is a so-called candidate cell state. Finally, ⊙ represents a multiply operation of the corresponding elements of the matrix.

### C. Support vector machine

SVM is a classic model that can be used not only for classification but also for regression. We do not delve into its theoretical derivation here, however. Unlike with a classification problem, the output of the regression problem is no longer a discrete value, but a continuous value. In reality, it is often impossible to accurately predict the value of the COVID-19, and to this end, it is particularly important to predict the development trend and change space for the important parameters of this disease.

Without considering other factors, time is clearly an important independent variable affecting COVID-19. To more accurately predict the change space of each group of data on this disease, in this paper, a fuzzy granulation model is introduced. First, we divide the original data into multiple granulation windows and then apply fuzziness to each window.

The so-called fuzzy granulation means that the information granulation adopted is based on the model of fuzzy set theory. The key to fuzzy granulation is fuzzification, here, we use the Pedrycz’s granulation method [33]. For a given time series, we consider the entire time series *X* as a window and then apply fuzziness to it. Fuzzification is a task used to establish a fuzzy particle *P* on *X*, that is, a fuzzy concept *G* that can reasonably describe *X*. Fuzzy particles can reasonably represent the original data and have certain specialties. A fuzzy particle *P* can be described simply as *P* = *A*(*x*), where *A* is a membership function of the fuzzy concept *G*. Commonly used fuzzy particles include triangular, ladder, Gaussian, and parabolic particles. In this study, we choose triangular fuzzy particles, the membership function of which is as follows:

$$A(x,a,m,b)=\left\{ \begin{array}{l} 0,x<a\\ \frac{x-a}{m-a},a\leq x\leq m\\ \frac{b-x}{b-m},m<x\leq b\\ 0,x>b \end{array}\right.$$

Here, *m* is the kernel of triangular fuzzy particles, and *a* and *b* are the lower and upper bounds of the support, respectively.

## Results

**Fig 3:**
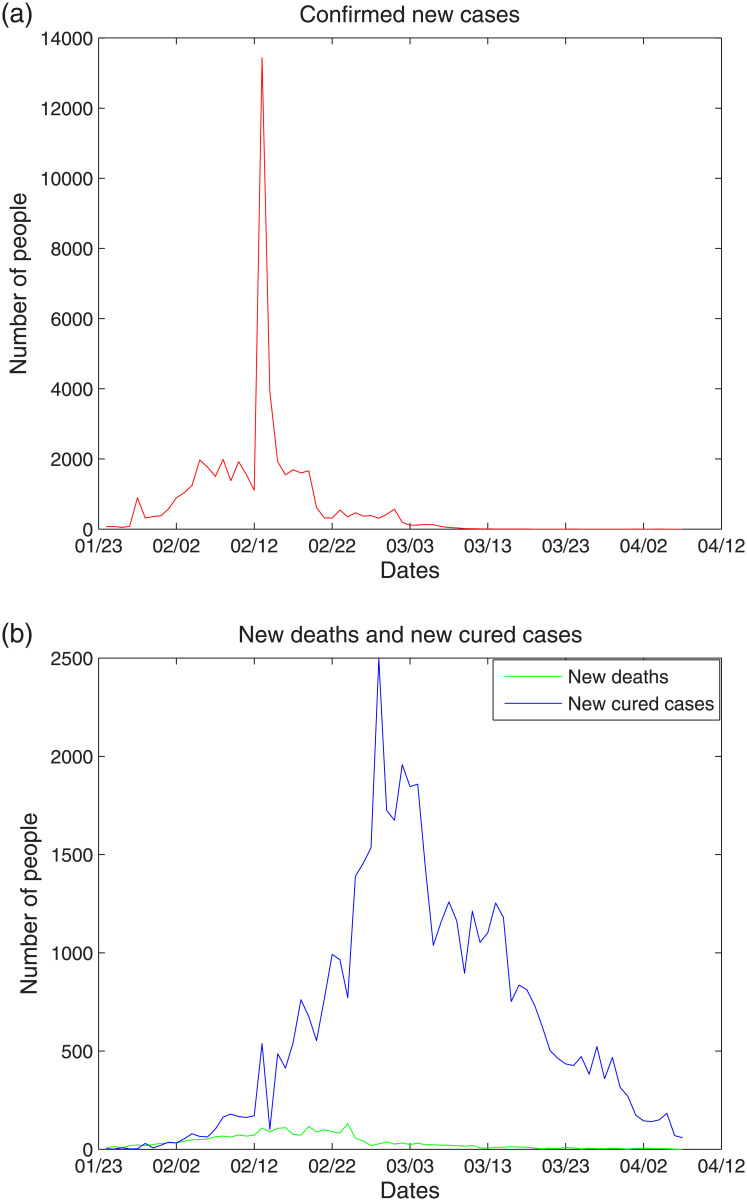
(a) The line chart of confirmed new cases. (b) The line chart of new deaths and new cured cases.

We studied COVID-19 data from January 23, 2020 to April 6, 2020, which were published by the Wuhan Municipal Health Commission (http://wjw.wh.gov.cn/) [34]. We used the Elman neural network, LSTM, and SVM to predict and analyze the development trend of cumulative confirmed cases, cumulative deaths, and cumulative cured cases. As an additional study, we also used the SVM with fuzzy granulation to predict and analyze the growth range of confirmed new cases, new deaths, and new cured cases. To further demonstrate the robustness of the models, we used the same models on data from the United States from March 23, 2020 to June 5, 2020. The data were obtained from the World Health Organization (WHO) [35]. The data from the United States contains confirmed cases, deaths while without cured cases. All experiments were conducted in MATLAB (R2014a). Fig 3(a) shows a line chart of the confirmed new cases. From Fig 3(a), it can be seen that, from January 23, 2020 to February 11, 2020, the number of confirmed new cases showed a fluctuating upward trend. confirmed new cases fluctuated sharply on February 12, showing a peak, the reason for which was the announcement of 12,364 clinically confirmed cases for the first time on that day, with a sharp increase in confirmed new cases. Fig 3(b) shows a line chart of the new deaths and cured cases. The number of new cured cases showed an increasing trend before February 27, after which the growth trend of the cured cases slowed. Moreover, the number of new deaths showed a significant downward trend after February 24, demonstrating a promising future recovery.

### A. Development trend prediction for data of Wuhan

In this section, the Elman neural network, LSTM, and SVM were used to predict and analyze the development trends of the cumulative confirmed cases, deaths, and cured cases. In the Elman neural network and LSTM, the experimental datas are in the form of the first three days to the fourth (i.e., using data from the first three days to predict the data on day four). Thus, the original 75 groups of data are converted into 72 groups, and the last group of data are used as the test sample. According to the data, both the Elman neural network and the LSTM in this paper contain only one hidden layer.

**Fig 4:**
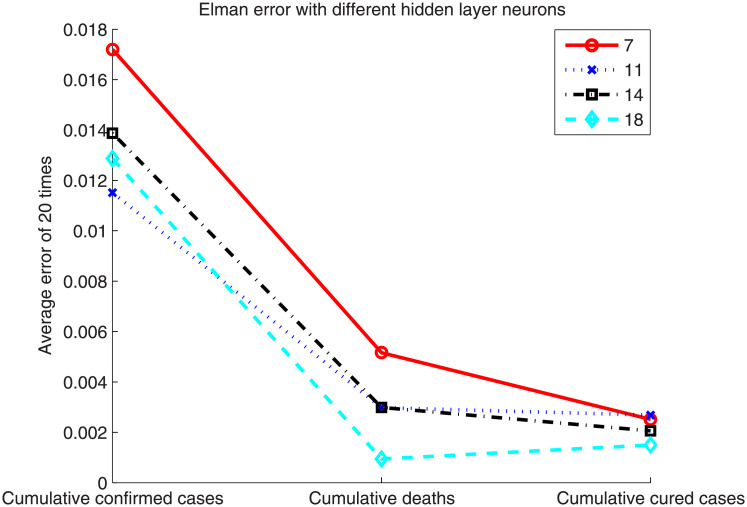
Error of the Elman neural network with different number of hidden layer neurons.

Because the initial weights of the Elman neural network and LSTM were randomly initialized, the average of 20 experimental results is given here as the final outcome. In addition, the number of hidden layer neurons is an important factor. We chose 7, 11, 14, and 18 hidden layer neurons according to experience. Fig 4 shows the error in the Elman neural network based on these different numbers of hidden layer neurons.

**Fig 5:**
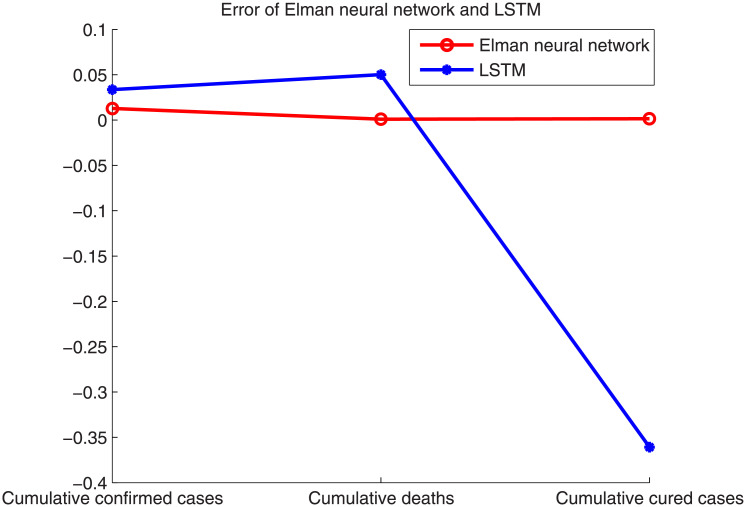
Error of the Elman neural network and LSTM.

Fig 4 shows that the effect of the number of hidden neurons on the error is not too obvious. By comparison, with 18 hidden neurons, the prediction result is slightly better. In addition, the prediction error of the cumulative confirmed cases is larger than that of the cumulative deaths and cumulative cured cases because the number of confirmed cases increased dramatically owing to the clinically confirmed cases announced on February 12, which has had a significant impact on the prediction results. Fig 5 shows the error rates of the Elman neural network and LSTM when the number of hidden layer neurons is 18. The prediction errors of the two methods are listed in Table 1.

**Table 1:** Error of the Elman neural network and LSTM for data of Wuhan.

Methods	Cumulative confirmed cases	Cumulative deaths	Cumulative cured cases
Elman neural network	0.0129	0.0009	0.0015
LSTM	0.0336	0.0501	-0.3609

As can be seen from Fig 5 and Table 1, the prediction results of the LSTM are worse than those of the Elman neural network, particularly for cumulative cured cases. As is well-known, LSTM is a powerful model for time-series prediction. However, the novel coronavirus pneumonia data have no obvious periodicity, and LSTM has a relatively poor prediction effect on data with a weak periodicity. Regarding the poor result of the cumulative cured cases, no effective treatment has been found, and fewer people were cured during the initial stage of the pandemic. In addition, it takes time between treatment and recovery. The treatment period varies from person to person. With continuous research, the number of cured cases continues to increase nonlinearly. For irregular growth, neural networks tend to fall into the local optimum, making it difficult to achieve an accurate prediction, which is also a major drawback of a neural network.

**Fig 6:**
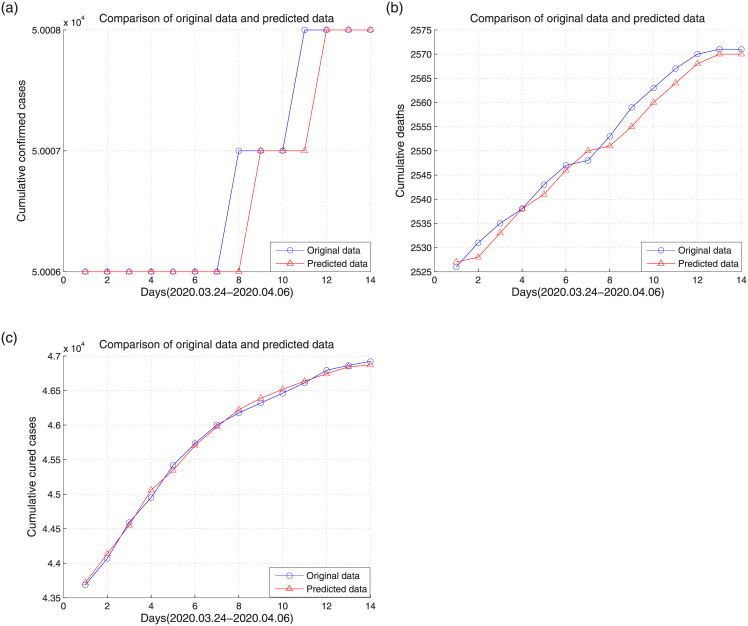
Prediction results of the cumulative confirmed cases, deaths and cured cases. (a) cumulative confirmed cases, (b) cumulative deaths, (c) cumulative cured cases.

In the SVM experiment, we used a one-day-to-the-next approach, that is, we used the data from the previous day to predict data for the next day. In this study, data of 61 days from January 23 to March 23 were used as training samples, and data from March 24 to April 6 were used as test samples. The radial basis function was used in the experiments. Fig 6 shows the prediction results of the cumulative confirmed cases, deaths, and cured cases.

The prediction mean square error and square correlation coefficient of the cumulative confirmed cases, deaths, and cured cases are shown in Table 2.

**Table 2:** Mean square error and square correlation coefficient for data of Wuhan.

Evaluation criterion	Cumulative confirmed cases	Cumulative deaths	Cumulative cured cases
Mean square error	0.0320	0.0023	0.0003
Square correlation coefficient	84.51%	99.06%	99.74%

It can be seen from the results that the SVM can predict the three sets of data well, and the square correlation coefficients of the cumulative deaths and cumulative cured cases between the predicted output and the actual output are all above 99%. Although the mean square error of the cumulative confirmed cases is relatively large and the square correlation coefficient is relatively small, it can be seen from Fig 6(a) that the fluctuation range of the cumulative confirmed cases from March 24 to April 6 is extremely small, and only two of the 14-day prediction results are incorrect.

### B. Development trend prediction for data of the United States

Similar to the aforedescribed experiments, herein we predict and analyze the development trend of cumulative confirmed cases and deaths from the data in the United States. Here, 12 hidden layer neurons of the Elman neural network and LSTM are applied. Table 3 shows the prediction error of the two methods on data from the United States.

**Table 3:** Error of the Elman neural network and LSTM for data of the United States.

Methods	Cumulative confirmed cases	Cumulative deaths
Elman neural network (18)	-0.0049	-0.0011
LSTM (18)	-0.1277	0.1118

**Fig 7:**
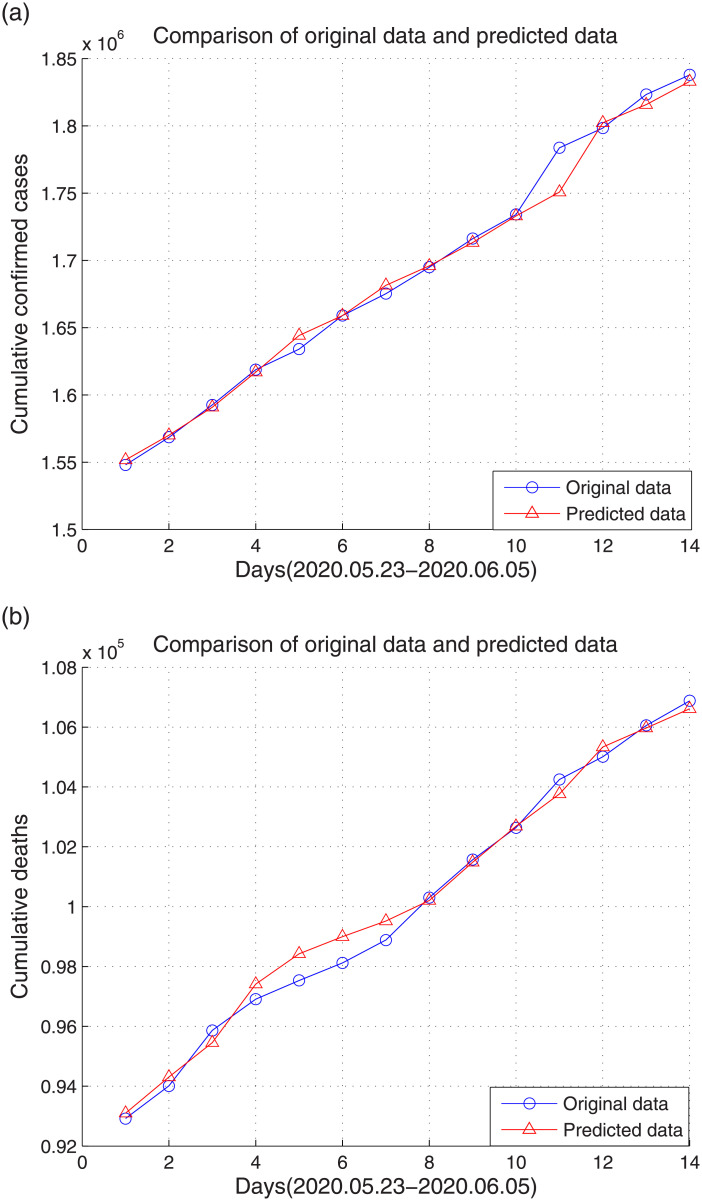
Prediction results of the cumulative confirmed cases and deaths. (a) cumulative confirmed cases, (b) cumulative deaths.

In the SVM experiment, data of 61 days from March 23 to May 22 were used as training samples, and data from May 23 to June 5 were used as test samples. The prediction results of the cumulative confirmed cases and deaths are shown in Fig 7. The mean square error and square correlation coefficient of the cumulative confirmed cases and deaths are shown in Table 4.

**Table 4:** Mean square error and square correlation coefficient for data of the United States.

Evaluation criterion	Cumulative confirmed cases	Cumulative deaths
Mean square error	0.0011	0.0011
Square correlation coefficient	99.03%	99.12%

Comparing the experimental results of Wuhan and the United States, the Elman neural network and SVM can well predict the development trend of Wuhan and the United States, whereas the prediction result of LSTM for the United States is clearly worse than that of Wuhan. This occurs because the data from the United States used in this study are national data, not data from a certain city, and the data growth is more irregular and without any periodicity.

### C. Growth range prediction for data of Wuhan

In addition to predicting the development trend of COVID-19, we also used a SVM with fuzzy granulation to predict the growth range of the confirmed new cases, new deaths, and new cured cases. Taking 5 days as a granulation window, the data from January 23, 2020 to April 1, 2020 were used as the training samples, and the growth range of the next 5 days was predicted, that is, the growth range of the confirmed new cases, new deaths, and new cured cases from April 2 to April 6. To reduce the impact of the peak value on the experimental results, we first smoothed the data of confirmed new cases and new cured cases. Table 5 shows the predicted growth range of confirmed new cases, new deaths, and new cured cases.

**Table 5:** Predicted growth range of the confirmed new cases, new deaths and new cured cases for data of Wuhan.

Dates	Actual growth					Predicted growth range	Predicted average
	April 2	April 3	April 4	April 5	April 6		
Confirmed new cases	0	1	0	0	0	[0,260]	160
New deaths	4	4	3	1	0	[4,10]	8
New cured cases	141	150	183	69	59	[24,498]	222

Table 5 shows that the growth ranges predicted in this paper are basically reasonable, although the growth range of new deaths is slightly large. However, the average predicted values are larger than actual average values. This is because the algorithm depends on the data, regardless of the actual situation. Owing to continuous medical research and strict control, there are increasingly fewer confirmed new cases and existing confirmed cases in Wuhan, resulting in increasingly fewer new deaths and cured cases. In the middle of the epidemic, the numbers of confirmed new cases and new cured cases increased rapidly, leading to a higher predicted average value. In addition, the number of confirmed cases had steadily increased from January 23 to February 11. However, on February 12, 12,364 clinically confirmed cases were announced for the first time and included in the confirmed new cases. The subsequent growth trends were completely inconsistent, which severely affected the prediction results.

### D. Growth range prediction for data of the United States

The data from March 23, 2020 to May 31, 2020 were used as the training samples, and the growth range of the confirmed new cases and new deaths from June 1 to June 5 were predicted. Table 6 shows the results.

**Table 6:** Predicted growth range of confirmed new cases and new deaths for data of the United States.

Dates	Actual growth					Predicted growth range	Predicted average
	June 1	June 2	June 3	June 4	June 5		
Confirmed new cases	17962	26116	14692	24890	14583	[18761,21027]	20045
New dead cases	1073	693	761	1043	825	[752,926]	813

The predicted growth range of confirmed new cases and new deaths is smaller than the actual growth. The predicted average for confirmed new cases is larger than the actual average of 19,648.6, whereas the predicted average for new deaths is smaller than the actual average of 879. The negative value in the public data has a significant influence on the prediction results (the number of confirmed new cases on May 10 is −99, and the number of new deaths on May 4 is −1696). There is no obvious downward trend in confirmed new cases and new deaths, and strict control is still required. Individuals should therefore strengthen their awareness of prevention and control and be responsible for themselves and others.

## Conclusions and future perspectives

The emergence of COVID-19 has been a heavy burden. During the past 2 months, however, significant progress has been made in the diagnosis and treatment of this disease. In this study, machine learning was used to predict and analyze the development trend and growth range of COVID-19. The results using data from Wuhan showed that the Elman neural network and SVM can better predict the development trend of cumulative deaths and cumulative cured cases, whereas the prediction error of cumulative confirmed cases is relatively large. In comparison, the LSTM model has a worse predictive effect on cumulative confirmed cases, deaths, and cured cases owing to the aperiodic data. Moreover, the prediction results of the two recurrent neural network models used in this study are unstable because neural networks tend to fall into a local optimum for data with irregular growth. This is a major drawback of neural networks, and needs to be improved in the future. For the prediction of the growth range of confirmed new cases, new deaths, and new cured cases, the SVM with fuzzy granulation introduced in this paper was shown to be effective. However, the predicted average values are larger than the actual average values, which indicates that the model is less robust to data with large fluctuations and still needs to be improved. The experimental results on data from the United States verified the robustness of the models, although the prediction result of LSTM was worse than that of Wuhan. In future research, we will improve the models based on the aforementioned problems and continue to improve the generalizability of the models.

## Supporting information

S1 Data Data of the United States. (XLSX)

S2 Data Data of Wuhan. (XLSX)

S1 File Data description. (DOCX)

S2 File Manuscript with marks. (DOCX)
