# Supplementary material for: Prediction and analysis of Corona Virus Disease 2019
Source: PLoS One. 2020 Oct 5;15(10):e0239960. doi: 10.1371/journal.pone.0239960 (PMC7535054; doi:10.1371/journal.pone.0239960)
Supplement: S1 File — (DOCX) [file pone.0239960.s003.docx]

**Data description**

The data mentioned in the Abstract and Conclusion refer to Data of Wuhan-Wuhan, Data of the United States-the United States;

In the part of Result A, C, Data of Wuhan / Data of the United States-Elman neural network and LSTM were used in the experiment of Elman neural network and LSTM; Data of Wuhan / Data of the United States-SVM were used in the experiment of SVM;

In the part of Result B, D, Data of Wuhan/Data of the United States-SVM with fuzzy granulation were used.

Note: The red ones are the training samples, and the blue ones are the test samples.
